# Postoperative fever after elective minimally invasive resection for gastric and colorectal cancer: incidence, risk factors and characteristics

**DOI:** 10.3389/fonc.2024.1413041

**Published:** 2025-01-10

**Authors:** Fan He, Chenglin Tang, Fuyu Yang, Dongqin Zhao, Junjie Xiong, Yu Zou, Defei Chen, Guoquan Huang, Kun Qian

**Affiliations:** ^1^ Department of Gastrointestinal Surgery, The First Affiliated Hospital of Chongqing Medical University, Chongqing, China; ^2^ Hubei Provincial Key Lab of Selenium Resources and Bioapplications, Enshi, China

**Keywords:** postoperative fever, gastrointestinal cancer, anastomotic leakage, multivariate analysis, retrospective studies

## Abstract

**Purpose:**

To analyze the incidence and risk factors of postoperative fever (POF) in gastrointestinal cancer (GIC), discuss the influence of POF on short-term clinical outcomes, and predict anastomotic leakage (AL) based on POF characteristics.

**Methods:**

Overall, 1362 patients that underwent radical resection for GIC were retrospectively analyzed. POF was defined as a postoperative temperature ≥38°C during hospitalization. Patients were divided according to whether they experienced POF. The influence of POF on short-term clinical outcomes was analyzed using propensity score matching. A subgroup analysis was conducted to examine the relationship between different POF characteristics and AL or infection-related complications.

**Results:**

POF occurred in 172 patients (12.6%). Overall, 115 patients (66.9%) had fever ≥38.6°C, while 105 (61.0%) had fever at postoperative day (POD) 2, and 73 (42.4%) had POF multiple times. Multivariate analysis showed that patients with a preoperative albumin level < 37 g/L (odds ratio [OR]=1.57, p=0.016), operative time >195min (OR=1.55, p=0.020), and radical gastrectomy (OR=1.84, p=0.009) were more likely to develop POF. Compared to patients without fever, drainage tube indwelling time, duration of antibiotic use, and hospital stay were prolonged, while AL and infection-related complications were more common in patients with POF. POF ≥38.6°C (OR=1.74, p=0.039) and PCT >0.7 ng/mL (OR=2.99, p=0.022) at POD 3 were early predictors of AL.

**Conclusion:**

POF was closely related to preoperative albumin levels, operative time, and type of operation, and it delayed postoperative recovery in patients with GIC. And POF ≥38.6°C and PCT >0.7 ng/mL at POD 3 were independent predictors of AL.

## 1 Introduction

Currently, radical resection of gastrointestinal cancer (GIC) is the preferred treatment for resectable gastrointestinal tumors. With the development of laparoscopic and robot-assisted surgical technology, radical resection of GIC is now considered to be very safe (1–3). However, some complications remain. The most common complication is postoperative fever (POF) (4) while the most serious complication is anastomotic leakage (AL). Previous studies (5–8) have found that POF is a common postoperative complication of major surgeries, such as abdominal or pelvic surgery, with an incidence of approximately 13%–50% depending on the surgical site and type of surgery. Besides, POF is more common in emergency abdominal surgeries (9). POF is usually divided into infectious or non-infectious fevers, and low-grade fever in the short term after surgery is often due to unknown causes. Most instances of POF are considered to be caused by non-infectious absorption of heat after surgery (10) and often does not draw the attention of surgeons. High POF and persistent fevers that are difficult to control are serious concerns for surgeons. For a gastrointestinal surgeon, the immediate reason for paying more attention to POF is fear of AL, which can be a devastating blow to patients undergoing digestive tract reconstruction (11).

However, most surgeons remain conservative regarding whether perioperative clinical management decisions need to be changed and whether early measures need to be taken to prevent serious complications of POF. Consequently, the current extensive tests for POF are less effective in identifying infection-related complications (6). The study by de la Torre et al. (12) showed that the clinical yield of most fever tests is low, and only patients with infection-related characteristics identified early are likely to benefit from laboratory and/or radiological tests for POF. To our knowledge, this is the first study to examine the effects of POF and its characteristics on the short-term clinical outcomes of GIC.

This study aimed to analyze the risk factors related to POF after elective gastrointestinal surgery, explore the impact of POF on short-term clinical outcomes, and identify AL early according to the characteristics of POF to guide the perioperative management of patients.

## 2 Methods

### 2.1 Patients

This study retrospectively analyzed patients diagnosed with gastric cancer (GC) and colorectal cancer (CRC) that underwent radical resection at the Department of Gastrointestinal Surgery, First Affiliated Hospital of Chongqing Medical University, from June 2022 to August 2023. All patients underwent standard radical tumor resection. This retrospective study did not require informed patient consent and was approved by the hospital’s ethics committee (Ethical ID: K2024-014-01).

The inclusion criteria required that patients were ≥ 18 years old and that GC and CRC were confirmed via pathological examination.

The exclusion criteria were as follows: 1. Patients who underwent emergency surgery, 2. Patients whose body temperature were ≥38°C within preoperative one week, 3. Patients who underwent laparotomy, 4. Patients who underwent palliative tumor resection, and 4. Patients who underwent complicated multivisceral resections.

### 2.2 Surgical procedures and perioperative management

Radical gastrectomy included laparoscopic distal or total gastrectomy, while radical resection of CRC included laparoscopic or robotic-assisted right hemicolectomy, laparoscopic or robotic-assisted left hemicolectomy, laparoscopic or robotic-assisted sigmoidoscopy, and laparoscopic or robotic-assisted radical resection of rectal cancer (RC). All radical tumor surgeries were performed by experienced associate director or above surgeons at the center, and the surgical procedures have been described previously (13–17). Ceftriaxone was administered intravenously as a prophylactic antibiotic (or levofloxacin in patients with a ceftriaxone allergy), and at least four ceftriaxone doses were administered to each patient (one during the surgery and again later in the same day, and two on postoperative day (POD) 1). All patients underwent colorectal surgery wound be orally prepared with a compound polyethylene glycol solution at the preoperative day to bowel preparation. An intraperitoneal drainage tube and catheter were routinely inserted during the operation, and the tube was removed postoperatively according to the patient’s condition. The nurses recorded the patient’s temperature at least four times a day (06:00, 14:00, 18:00, and 22:00) and repeated measurements were performed for those with abnormal body temperatures. To consider the possibility of infection-related complications, surgeons selectively arranged relevant examinations, including abdominal computed tomography (CT), blood culture, drainage tube culture, and sputum culture. All patients received prophylaxis for deep vein thrombosis (subcutaneous injection of low-molecular-weight heparin) on POD 2. After surgery, anisodamine and flurbiprofen were selectively applied to the pain, and patients with high POF that were difficult to control were administered aminobarbital to reduce fever.

### 2.3 Definition

POF was defined as a postoperative body temperature ≥ 38°C during hospitalization (5, 12, 18). The patients were divided into fever and no-fever groups according to whether they had POF. In the fever group, fever characteristics, including the time of initial fever, extent of fever, and frequency of fever, were analyzed. Chronic pulmonary disease was defined as a history of chronic obstructive pulmonary disease, chronic cough and sputum production, chronic asthma, and tuberculosis. An abdominal infection was defined as a patient presenting signs of peritonitis with turbid drainage from the abdominal drainage tube or an abdominal CT examination indicating an abdominal infection or a positive bacterial culture from the abdominal drainage tube (19). Pulmonary infection was defined as persistent cough with yellowish-green sputum, a positive bacterial culture of sputum, or chest CT indicating a pulmonary infection (20). AL was defined as a defect in the intestinal wall at the anastomotic site resulting in internal and external communication in the intestinal cavity. The clinical manifestations of AL are usually shown by upper digestive tract angiography, AL indicated by abdominal CT, or drainage of fecal-like fluid from an abdominal drainage tube (21). Surgical site infection was defined as incision rupture or purulent discharge in patients (20). According to the optimal cutoff value, postoperative leukocytosis was defined as a 70% increase in white blood cell (WBC) count on POD 3 compared with the preoperative value.

### 2.4 Statistical analysis

SPSS version 27.0 (IBM Corp., Armonk, NY, USA) was used for the statistical analyses. GraphPad Prism software (version 9.0) was used to edit statistical graphs. P<0.05 was considered statistically significant. Continuous variables with a normal distribution are represented as means and standard deviations. Student’s *t*-tests were used to compare differences. Continuous variables with non-normal distributions were represented as medians and quartiles, and the Mann-Whitney U test was used to compare the differences. Categorical variables were expressed as frequency (%), and differences were compared using the chi-square test and Fisher’s exact test. Continuity variables were dichotomised for disease risk differentiation and clinical decision-making, and the best truncation values of the continuity variables were obtained using receiver operating characteristic curve analysis. The influence of POF on short-term postoperative clinical outcomes was analyzed using propensity score matching (PSM) to exclude preoperative confounding factors. Univariate and multivariate analyses were used to determine the risk factors for POF, and the risk factors for AL were determined and represented as an odds ratio (OR) and 95% confidence interval (CI).

## 3 Results

**Figure 1:**
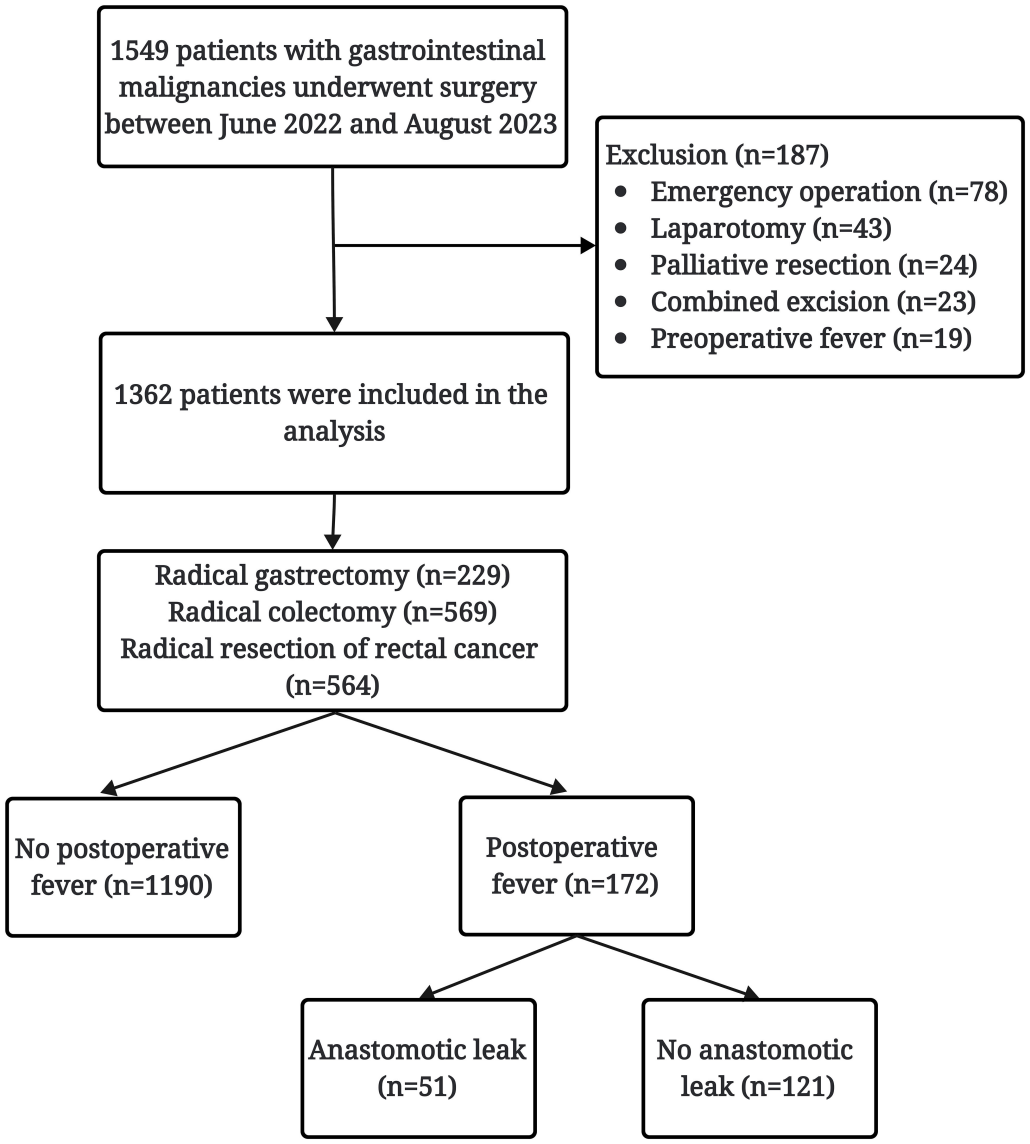
Flow chart of the patients included in this study.

During the study period, 1549 patients with GIC underwent radical resection in our department, of which 78 underwent emergency surgery, 43 underwent laparotomy, 24 underwent palliative resection, and 23 underwent multivisceral resection, and 19 exhibiting preoperative fever were excluded. Overall, 1362 patients were included in the final analysis. Among these patients, 229 underwent radical resection for GC, 569 underwent radical resection for colon cancer, and 564 underwent radical resection for RC (**Figure 1**). The demographic data of the included patients and preoperative laboratory results are shown in **Supplementary Table 1**.

### 3.1 Incidence of POF

**Figure 2:**
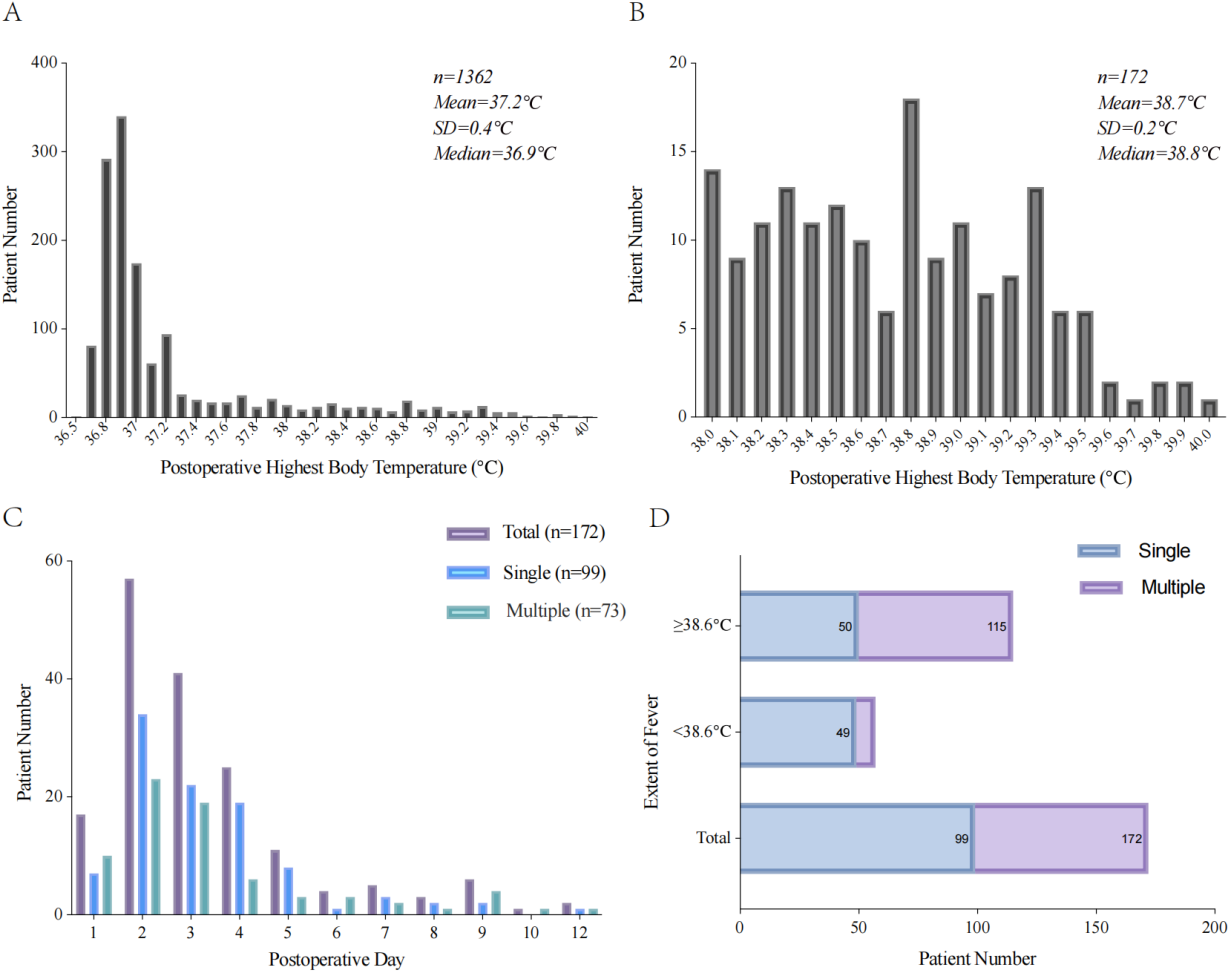
Characteristics of POF with patient. **(A)** Histogram of postoperative highest body temperature. **(B)** Histogram of postoperative highest body temperature with POF. **(C)** The time of first fever after radical resection of GIC. **(D)** The frequency of POF according to the extent of maximum temperature.

Among the 1362 patients with GIC, 172 (12.6%) experienced POF. The average body temperature of the patients was 37.2°C, and the temperature of most patients was <37°C. In patients with fever, the average body temperature was 38.7°C. In addition, 67 (39.0%) patients began to develop fever in POD <2, 115 (66.9%) had POF with a maximum temperature of ≥38.6°C, and 73 (42.4%) had multiple POF. Among the patients with postoperative body temperature ≥38.6°C, 65 (37.8%) had multiple POF; in contrast, among the patients with body temperature <38.6°C, only 8 (14.0%) had multiple POF (**Figure 2**).

### 3.2 Risk factors for POF

A univariate analysis identified 11 factors associated with POF. Among these factors, patients that were ≥70 years old (OR=1.452, p=0.026), male (OR=1.615, p=0.007), had an ASA score ≥3 (OR=1.481, p=0.016), underwent surgery for GC (OR=2.336, p <0.001), had an age-adjusted Charlson Comorbidity Index (aCCI) >5 (OR=1.527, p=0.011), were smokers (OR=1.404, p=0.047), had chronic pulmonary disease (OR=1.713, p=0.009), had an operative time ≥195min (OR=1.725, p=0.001), experienced intraoperative blood loss >50 mL (OR=1.531, p=0.009), had preoperative WBC <6.5 × 10^9^/L (OR=1.646, p=0.009), or had preoperative albumin <37 g/L (OR=1.771, p=0.001) were correlated with POF. Multivariate analysis showed that when compared with patients with RC, patients with GC were more likely to develop POF (OR=1.839, 95%CI 1.167-2.899, p=0.009). Additionally, an operative time ≥195min (OR=1.552, 95%CI 1.073-2.245, p=0.020) and preoperative albumin <37 g/L (OR=1.565, 95%CI 1.085-2.256, p=0.016) were independent risk factors for POF in patients with GIC (**Table 1**).

**Table 1:** Risk factors for the occurrence of postoperative fever.

Variables	Univariate		Multivariate	
	OR(95% CI)	p	OR(95% CI)	p
**Age (≥70 vs <70) (years)**	1.452 (1.046~2.014)	0.026	1.309 (0.820~2.091)	0.260
**Sex (male vs female)**	1.615 (1.139~2.290)	0.007	1.407 (0.938~2.112)	0.099
**BMI (≥25 vs <25) (kg/m^2^)**	1.063 (0.729~1.549)	0.751		
**ASA score (3 vs 1/2)**	1.481 (1.075~2.041)	0.016	0.945 (0.486~1.840)	0.869
**Tumor type**				
Colon cancer vs gastric cancer	0.500 (0.332~0.752)	0.001	0.678 (0.429~1.070)	0.095
Gastric cancer vs rectal cancer	2.336 (1.538~3.550)	<0.001	1.839 (1.167~2.899)	0.009
**aCCI (>5 vs ≤5)**	1.527 (1.100~2.119)	0.011	1.227 (0.682~2.208)	0.495
**Smoking (yes vs no)**	1.404 (1.005~1.962)	0.047	1.205 (0.816~1.778)	0.349
**Alcohol consumption (yes vs no)**	1.300 (0.908~1.863)	0.152		
**Hypertension (yes vs no)**	1.188 (0.838~1.686)	0.334		
**Diabetes (yes vs no)**	0.986 (0.622~1.564)	0.954		
**Chronic pulmonary disease (yes vs no)**	1.713 (1.144~2.565)	0.009	1.459 (0.936~2.275)	0.096
**Coronary artery disease (yes vs no)**	1.216 (0.659~2.241)	0.532		
**Metastasis (yes vs no)**	1.162 (0.993~1.361)	0.062		
**NRT (yes vs no)**	1.041 (0.555~1.952)	0.901		
**NCT (yes vs no)**	1.328 (0.856~2.060)	0.206		
**Operative time (≥195 vs <195) (min)**	1.725 (1.242~2.397)	0.001	1.552 (1.073~2.245)	0.020
**Blood loss (>50 vs ≤50) (ml)**	1.531 (1.110~2.111)	0.009	0.977 (0.682~1.399)	0.899
**Hb (≥100 vs <100) (g/L)**	0.730 (0.495~1.079)	0.114		
**WBC (<6.5 vs ≥6.5) (10^9^/L)**	1.646 (1.133~2.392)	0.009	1.324 (0.890~1.970)	0.166
**Alb (≤37 vs >37) (g/L)**	1.771 (1.248~2.512)	0.001	1.565 (1.085~2.256)	0.016
**IL-6 (≥6 vs <6) (pg/ml)**	0.994 (0.975~1.014)	0.562		

OR, Odds Ratio; CI, Confidence Interval; aCCI, Age-adjust Charlson Comorbidity Index; NRT, Neoadjuvant Radiotherapy; NCT, Neoadjuvant Chemotherapy; Hb, Hemoglobin; WBC, White Blood Cell; Alb, albumin; IL-6, interleukin-6.

### 3.3 Influence of POF

Patients with POF were matched 1:1 with those without POF according to the risk factors for POF. The matching results included 163 patients in the fever and no-fever groups. Demographic data of the patients after PSM are shown in **Supplementary Table 2**. Comparing the short-term clinical outcomes of POF for GIC, patients with POF had higher WBC and PCT counts at POD 3 (p=0.008 and p<0.001, respectively), longer drainage tube indwelling time (p=0.046), catheter indwelling time (p=0.003), antibiotic use duration (p<0.001), and hospital stays (p<0.001). Postoperative infection-related complications (p<0.001) and hospitalization costs were also higher (p<0.001). No significant differences in the time to first feeding, postoperative pain, readmission, or reoperation (all p>0.05) were observed. In addition, we found that the positive rates of blood and sputum cultures in the fever group were lower than those without fever (9.0% vs 37.5%, p=0.042), the positive rates of drainage tube culture were higher than those in the group without fever (53.3% vs 20.8%, p<0.001), and there was no significant difference in the positive rate of sputum culture between the two groups (19.4% vs 40%, p=0.125) (**Table 2**).

**Table 2:** Influence of postoperative fever on clinical outcome compared with no fever.

Variables	Fever (n=163)	No Fever (n=163)	p
**WBC of POD1 (10^9^/L)**	10.24(8.43-12.76)	10.22(8.33-12.18)	0.694
**PCT of POD1 (ng/ml)**	0.19(0.08-0.47)	0.17(0.07-0.29)	0.216
**CPR of POD1 (mg/L)**	28.70(14.59-62.35)	31.00(12.50-72.65)	0.498
**WBC of POD3 (10^9^/L)**	8.92(6.93-11.17)	7.81(6.51-9.84)	0.008
**PCT of POD3 (ng/ml)**	0.46(0.28-0.90)	0.23(0.10-0.44)	<0.001
**CPR of POD3 (mg/L)**	104.00(77.30-181.00)	93.80(65.80-149.50)	0.551
**Duration of antibiotic use (day)**	9 (5–13)	2 (2–6)	<0.001
**Drainage tube indwelling time (day)**	9 (9–15)	7 (6–8)	0.046
**Catheter indwelling time (day)**	4 (2–5)	5 (2–8)	0.003
**Hospital stay (day)**	11 (9–15)	7 (6–9)	<0.001
**Time of first defecation (day)**	4 (3–6)	4 (3–5)	0.552
**Time of first feeding (day)**	4 (3–6)	4 (3–7)	0.934
**Anisodamine is antispasmodic**	19(11.7%)	16(9.8%)	0.591
**Flurbiprofen exate labor pains**	0 (0–3)	0 (0–2)	0.689
**Blood bacterial culture**			0.042
(–)	92(90.0%)	5(62.5%)	
(+)	9(9.0%)	3(37.5%)	
**Drainage tube bacteria culture**			<0.001
(–)	43(46.7%)	38(79.2%)	
(+)	49(53.3%)	10(20.8%)	
**Sputum culture**			0.125
(–)	29(80.6%)	9(60%)	
(+)	7(19.4%)	6(40%)	
**Postoperative CT examination**	79(48.5%)	32(19.6%)	<0.001
**Anastomotic leakage**	49(30.1%)	4(2.5%)	<0.001
**Abdominal infection**	78(47.9%)	20(12.3%)	<0.001
**Pulmonary infection**	57(35.0%)	11(6.7%)	<0.001
**Postoperative ICU**	4(2.5%)	5(3.1%)	1.000
**Postoperative hemorrhage**	5(3.1%)	3(1.8%)	0.723
**Readmission**	6(3.7%)	1(0.6%)	0.121
**Reoperation**	5(3.1%)	2(1.2%)	0.448
**Hospital cost (yuan)**	75296.61(68279.61-88914.86)	66864.86(59339.61-73726.93)	<0.001

WBC, White Blood Cell; PCT, Procalcitonin; CPR, C-reactive Protein; POD, Postoperative day; CT, Computed Tomography; ICU, Intensive Care Unit.

### 3.4 Relationship between POF characteristics and AL or infection-related complications

Subgroup analysis indicated that the incidence of AL and abdominal infection in patients with POF ≥38.6°C was significantly higher than that in patients with POF <38.6°C (p=0.036), and both hospital stays and duration of antibiotic use were longer (p=0.025 and p=0.025). The differences in AL and infection-related complications between patients with fever in POD ≤ 2 and those with fever in POD>2 were not significantly different (p>0.05), while patients with fever in POD>2 had significantly longer postoperative hospital stays (p<0.001), duration of antibiotic use (p=0.008), and drainage tube indwelling time (p=0.02). Moreover, we found that patients with multiple POFs had a higher incidence of AL (p=0.032) and abdominal infection (p=0.041), while also experiencing longer postoperative hospital stays (p=0.002), duration of antibiotic use (p<0.001), and drainage tube indwelling times (p=0.021). Finally, hospitalization costs (p=0.008) were higher for patients with multiple POF than for patients with a single POF (p=0.008) (**Table 3**).

**Table 3:** Outcome of the 172 patients with POF according to the characteristics of fever.

Variables	Extent of fever			Time of first fever			Frequency of fever		
	38.0~38.6°C (n = 57)	>38.6°C (n = 115)	p	POD ≤ 2 (n= 67)	POD>2 (n=105)	p	Single (n = 99)	Multiple (n = 73)	p
**Anastomotic leakage**	11(19.3%)	40(34.8%)	0.036	15(22.4%)	36(34.3%)	0.096	23(23.2%)	28(38.4%)	0.032
**Abdominal infection**	20(35.1%)	61(53.0%)	0.026	26(38.8%)	55(52.4%)	0.082	41(41.1%)	40(54.8%)	0.041
**Pulmonary infection**	16(28.1%)	44(38.3%)	0.187	29(43.3%)	31(29.5%)	0.065	30(30.3%)	30(41.1%)	0.142
**Surgical site infection**	2(3.5%)	5(4.3%)	0.793	2(3.0%)	5(4.8%)	0.565	4(4.0%)	3(4.1%)	0.982
**Urinary tract infection**	1(1.8%)	1(0.9%)	1.000	1(1.5%)	1(1.0%)	1.000	2(2.0%)	0(0%)	0.509
**Bacterial culture (+)**	17(29.8%)	49(42.6%)	0.105	19(28.4%)	47(44.8%)	0.031	30(30.3%)	36(49.3%)	0.011
**Hospital stay (day)**	11 (8–13)	12 (9–15)	0.025	10 (8–12)	12 (10–19)	<0.001	11 (8–14)	12 (10–18)	0.002
**Hospital cost (yuan)**	70389 (65865–87233)	80216 (68383–92896)	0.125	72323 (66510–84688)	80266 (68383–95906)	0.099	71883 (67173–82813)	83126 (68603–97045)	0.008
**Postoperative ICU**	1(1.8%)	3(2.6%)	1.000	0(0%)	4(3.8%)	0.158	1(1.0%)	3(4.1%)	0.313
**Readmission**	1(1.8%)	5(4.3%)	0.665	4(6.0%)	2(2.0%)	0.210	4(4.0%)	2(2.7%)	1.000
**Reoperation**	2(3.5%)	3(2.6%)	1.000	1(1.5%)	4(3.8%)	0.650	2(2.0%)	3(4.1%)	0.652
**Mortality**	1(1.8%)	0(0%)	0.331	1(1.5%)	0(0%)	0.390	1(1.0%)	0(0%)	1.000
**Duration of antibiotic use (day)**	8.26 ± 5.06	10.46 ± 6.41	0.025	8.21 ± 4.51	10.7 ± 6.72	0.008	8.26 ± 5.06	10.46 ± 6.41	<0.001
**Drainage tube extraction time (day)**	9 (7–12)	10 (7–13)	0.219	8 (7–12)	10 (8–15)	0.020	9 (7–12)	11 (8–14)	0.021

POF, Postoperative Fever; POD, Postoperative day; ICU, Intensive Care Unit.

We also performed a subgroup analysis of patients with or without AL. Independent risk factors for AL were explored using POF characteristics and postoperative laboratory infection indicators. Univariate analysis showed that patients with POF ≥38.6°C (OR=1.49, p=0.036), multiple POF (OR=2.06, p=0.033), and PCT >0.7 ng/mL at POD 3 (OR=2.55, p=0.033) had a higher incidence of AL. Multivariate analysis showed that the POF ≥38.6°C (OR=1.73, 95%CI 1.03-2.95, p=0.039) and PCT >0.7 ng/mL at POD 3 (OR=2.99, 95%CI 1.17-7.68, p=0.022) were independent predictors of AL (**Table 4**).

**Table 4:** Predictive value of POF characteristics and postoperative laboratory infection indicators on postoperative AL.

Variables	Univariate		Multivariate	
	OR (95% CI)	p	OR (95% CI)	p
**Extent of fever (>38.6 vs ≤38.6˚C)**	1.49(1.02-2.19)	0.039	1.74(1.03-2.95)	0.039
**Time of first fever (POD >2 vs ≤ 2)**	1.81(0.90-3.56)	0.098	1.74(0.68-4.50)	0.247
**Frequency (multiple vs single)**	2.06(1.06-3.99)	0.033	1.40(0.54-3.58)	0.480
**Leukocytosis (yes or no)**	1.30(0.66-2.59)	0.449		
**PCT on POD 3(<0.7 ng/ml vs ≥0.7 ng/ml)**	2.55(1.08-6.02)	0.033	2.99(1.17-7.68)	0.022

POF, Postoperative Fever; AL, anastomotic leakage; OR, Odds Ratio; POD, Postoperative day; PCT, Procalcitonin.

## 4 Discussion

This study discovered that patients with an operative time >195min, preoperative albumin < 37 g/L, and those who underwent radical gastrectomy were more likely to develop POF. In these patients, the probability of AL and infection-related complications increased significantly, which extended the hospital stay, increased the hospitalization cost, and seriously affected the postoperative rehabilitation of patients. Additionally, we also found that patients with POF ≥38.6°C and PCT >0.7 ng/mL at POD 3 were more likely to develop AL after digestive tract reconstruction. This helps identify AL early and should reduce the occurrence of serious infections. In addition, 172 patients who underwent radical resection for GIC developed POF in this study, and the occurrence of POF was 12.6%, which was similar to the results of Booth et al. (5) which showed that 13.8% of patients developed POF after radical resection of CRC.

In this study, 51 (30.0%) patients’ POF were directly caused by AL, while 77 (44.8%) were caused by other infection-related complications, and the cause was unclear in 44 patients (25.6%). As the most concerning complication for gastrointestinal surgeons, AL attracts special attention in all patients with POF, including a series of examinations and treatments, such as abdominal CT, blood culture, drainage tube culture, abstinence from food and drink, and advanced antibiotics, which increase the economic and psychological burden on patients. This study analyzed, for the first time, the risk and characteristics of POF during radical resection of GIC as well as its relationship with AL or infection-related complications, providing a theoretical and practical basis for gastrointestinal surgeons to manage POF.

### 4.1 Incidence of POF

Patients whose operative time was >195min had a higher risk of POF, which is consistent with the findings of both Mayo et al. (22) and Nakanishi et al. (23). The underlying reason for this result can be explained. First, a longer operative time tends to lead to greater tissue destruction, and the release of inflammatory cytokines (such as interleukin 6) during tissue destruction is directly related to POF (24). A longer operative time also exposes patients to air for a long time, which increases the risk of infection. Second, a longer operative time means longer periods of anesthesia. Karam et al. (25) and Beilin et al. (26) have shown that the use of large doses of anesthetics increases the risk of POF. Anesthetics such as fentanyl inhibit natural killer cell-mediated cytotoxicity and may weaken the immune response, increasing the risk of POF and infectious complications. Guidolin et al. (27) found that a longer operative time is a risk factor for postoperative infection-related complications. Therefore, surgeons should shorten the operative time as much as possible on the premise of ensuring surgical quality.

Hypoproteinaemia has been shown in past studies to increase the risk of POF (28, 29), and this is consistent with the results from this study. Lower albumin levels lead to insufficient synthesis of immunoglobulins, reducing patients’ ability to fight infection. Conversely, insufficient albumin may reduce polyunsaturated fatty acid mobilization, reduce the formation of anti-inflammatory lipids (30), and weaken the anti-inflammatory abilities of patients. In addition, Kang et al. (31) found that preoperative hypoproteinemia significantly increased complications, such as abdominal and pulmonary infections, in patients with CRC after radical surgery. In this study, the risk of POF in patients with preoperative albumin < 37 g/L was 1.7-fold higher than that in patients with ≥ 37 g/L. For patients with known preoperative hypoproteinemia, we should actively improve their nutritional status or perform perioperative intravenous infusion of human albumin before performing radical tumor resection. In addition, the probability of POF in patients with GC included in the analysis was higher than that in patients with RC. However, no statistically significant difference in AL or abdominal infection was observed. This may be because radical gastrectomy involves more complicated surgical procedures, more trauma, longer operative time, more intraoperative bleeding, and more anastomosis after digestive tract reconstruction. Under the influence of these factors, the probability of POF in patients will increase.

### 4.2 Influence of POF

To analyze the impact of POF on the short-term clinical outcomes of patients, we conducted 1:1 PSM for patients with or without POF to increase the reliability of the results. This analysis showed that AL and infection-related complications were significantly more common in patients with POF. The duration of antibiotic use, indwelling time of the drainage tube, length of hospital stay, and auxiliary examinations for fever also increased. Among the patients with POF, more than half underwent blood culture, and the positivity rate was significantly lower than that of the group without POF (9.0%). For patients who have just undergone grade 4 surgery, frequent blood collection is not conducive to postoperative rehabilitation and increases the pain and economic burden on patients. However, no significant difference in the time of first feeding and defecation between the two groups was observed, indicating that clinicians failed to detect the occurrence of AL and infection-related complications in patients and instructed them to feed, which may have aggravated the symptoms of postoperative infection. In addition, approximately 30% of the examined patients had POF due to AL. Therefore, we should be vigilant about the diet of patients with POF to avoid increasing symptoms of infection. Gastrointestinal surgeons should strengthen perioperative management, control postoperative temperature, and take early measures to prevent high POF occurrence.

### 4.3 Relationship between POF characteristics and AL or infection-related complications

In a subgroup analysis, we found that AL and abdominal infection were more common in patients with POF ≥38.6°C and multiple POFs, while pulmonary infections were more common in patients with fever with POD <2. Patients with multiple POFs were more likely to have bacteriological evidence in the drainage tubes and blood, whereas patients with fever on POD <2 were more likely to have bacteriological evidence in the sputum. However, regardless of the POF characteristics, they all increased the duration of antibiotic use and hospital stays. Hospitalization costs were significantly higher in patients with multiple POFs than in patients with a single POF. During perioperative management, more attention should be paid to patients with multiple POFs and a high postoperative fever. Based on the characteristics of patients with POF, we can improve the necessary auxiliary examination and treatment, improve the rate of lesion detection, detect infected lesions early, and accelerate the postoperative rehabilitation of patients.

In our study, the incidence of AL in patients with POF was relatively high (approximately 30%), attracting the attention of surgeons. Therefore, we analyzed the characteristics of POF combined with postoperative infection indicators for the early identification of AL. The results showed that postoperative hyperthermia (body temperature ≥38.6°C) and PCT >0.7 ng/mL at POD 3 were independent predictors of AL in patients with POF. Previous studies showed similar results. A meta-analysis by Xu et al. (32) found that PCT levels on POD 3 contributed to the early diagnosis of AL after CRC surgery. The release of PCT is specifically induced by bacterial endotoxins and does not increase following noninfectious inflammation. In healthy individuals, the serum PCT concentration is <0.05 ng/mL. However, in response to bacterial infections, damage-associated molecular patterns and pathogen-associated molecular patterns stimulate cells to produce PCT, significantly increasing serum concentrations (33). To our surprise, CRP in patients with fever was higher than that in patients without fever on POD 3, but there was no statistically significant difference. Although CRP is a commonly used indicator of inflammation in clinical practice, its specificity is poor, and most patients underwent major abdominal surgery would have elevated CRP postoperatively. Therefore, PCT has a high specificity for the identification of abdominal infections and AL. After AL occurs, food residues and bacteria in the gastrointestinal tract are transferred to the abdomen, and many inflammatory factors are produced in the abdomen and other tissues, entering into the blood. Patients often present with a high POF and serious peritonitis, and some may even exhibit septic shock. Therefore, we should strengthen the perioperative management of patients with high POF and PCT levels, control the diet of patients and administration of antibiotics, and reduce the occurrence of serious complications. However, due to the excessive tension of some surgeons and patients’ families and the fear of AL, many unnecessary examinations and treatments have been performed, which has increased the economic burden on patients, relaxed the guidelines for the use of antibiotics, and increased the generation of drug-resistant bacteria. This is detrimental to patient management and the development of antibiotics.

### 4.4 Strengths and limitations

This study comprehensively analyzed the risk and incidence of POF in patients with GIC after radical surgery and provided theoretical guidance for gastrointestinal surgeons in preventing POF. Simultaneously, the serious influence of POF on postoperative rehabilitation was analyzed, attracting surgeons’ attention to POF. In addition, we analyzed the characteristics of POF for the first time, which changed the previous treatment method and provided a practical basis for the targeted treatment of POF in perioperative patients.

However, this study had some limitations. First, this was a single-center, retrospective study. Second, the patients underwent several types of operations. However, they all underwent radical resection of GIC and all were grade 4 major operations; therefore, we conducted a unified analysis. Third, because of the control of postoperative body temperature by clinicians, most patients experienced less POF; therefore, we failed to analyze the association between different fever types and infection-related complications. Finally, this study only analyzed the relationship between POF characteristics and AL or infection-related complications and failed to provide specific perioperative fever management plans. Because of insufficient follow-up time, we only analyzed the impact of POF on the short-term clinical outcomes of patients and failed to analyze the impact of POF on long-term prognosis. In future, we will continue to conduct rigorously designed randomized controlled trials to summarize and explore the effects of specific perioperative fever management and perform longer follow-up on the tumor prognosis of these patients.

## 5 Conclusion

This study found that 12.6% of the patients who underwent radical resection for GIC developed POF. Patients with low preoperative albumin level, long operative time, and radical resection of GC were more likely to develop POF. Additionally, more AL and infection-related complications in patients with POF were observed, which seriously affected postoperative rehabilitation. In addition, POF ≥38.6°C and PCT >0.7 ng/mL at POD 3 were independent predictors of AL. Based on the results of this study, surgeons can improve perioperative fever management to help patients recover postoperatively.

## Data Availability

The raw data supporting the conclusions of this article will be made available by the authors, without undue reservation.
