# Supplementary material for: Postoperative fever after elective minimally invasive resection for gastric and colorectal cancer: incidence, risk factors and characteristics
Source: Front Oncol. 2025 Jan 10;14:1413041. doi: 10.3389/fonc.2024.1413041 (PMC11759314; doi:10.3389/fonc.2024.1413041)
Supplement: Supplementary file 1 [file Table1.docx]

| **Supplementary Table 1: The main characteristics of the patients.** | |
| --- | --- |
| **Variables** | **Total (n=1362)** |
| **Age (years)** | 63.75±11.89 |
| **Male** | 838(61.53%) |
| **Body mass index (kg/m^2^)** | 22.93±3.25 |
| **ASA score** |  |
| 1 | 287(21.07%) |
| 2 | 494(36.27%) |
| 3 | 581(42.66%) |
| **Tumor type** |  |
| Gastric cancer | 229(16.81%) |
| Colon cancer | 564(41.41%) |
| Rectal cancer | 569(41.78%) |
| **Clinical stage** |  |
| Ⅰ | 352(25.84%) |
| Ⅱ | 533(39.13%) |
| Ⅲ | 415(30.47%) |
| Ⅳ | 62(4.6%) |
| **Age-adjust Charlson Comorbidity Index** | 5.02±1.28 |
| **Smoking** | 410(30.10%) |
| **Alcohol consumption** | 321(23.57%) |
| **Hypertension** | 370(27.17%) |
| **Diabetes** | 192(14.10%) |
| **Chronic pulmonary disease** | 195(14.32%) |
| **Coronary artery disease** | 88(6.46%) |
| **Neoadjuvant radiotherapy** | 92(6.75%) |
| **Neoadjuvant chemtherapy** | 180(13.22%) |
| **Operative time (min)** | 208.3±75.57 |
| **Intraoperative blood loss (ml)** | 79.9±97.41 |
| **Preoperative hemoglobin (g/L)** | 120.30±23.53 |
| **Preoperative white blood cell (10^9^/L)** | 5.68±1.89 |
| **Preoperative albumin (g/L)** | 37.09±11.45 |
| **Preoperative interleukin-6 (pg/ml)** | 8.77±24.75 |
| **Hospital costs (yuan)** | 67876.61±17547.91 |

ASA: American Society of Anesthesiologists

| **Supplementary Table 2: Baseline characteristics before and after PSM.** | | | | | | |
| --- | --- | --- | --- | --- | --- | --- |
|  | **Before PSM** | | | **After PSM** | | |
| **Variables** | **Fever(n=172)** | **No Fever(n=1190)** | **p** | **Fever (n=163)** | **No Fever (n=163)** | **p** |
| **Age (years)** | 65.74±11.42 | 63.46±11.94 | 0.019 | 65.39±11.37 | 65.84±11.37 | 0.719 |
| **Male** | 50(29.1%) | 474(39.8%) | 0.007 | 49(30.1%) | 49(30.1%) | 1 |
| **Body mass index (kg/m^2^)** | 22.87±3.59 | 22.94±3.20 | 0.795 | 22.82±3.64 | 22.79±3.40 | 0.943 |
| **ASA score** |  |  | 0.030 |  |  | 0.885 |
| 1 | 26(15.1%) | 261(21.9%) |  | 25(15.3%) | 26(16.0%) |  |
| 2 | 58(33.7%) | 436(36.6%) |  | 58(35.6%) | 54(33.1%) |  |
| 3 | 88(51.2%) | 493(41.4%) |  | 80(49.1%) | 82(50.3%) |  |
| **Tumor type** |  |  | <0.001 |  |  | 0.941 |
| Gastric cancer | 48(27.9%) | 181(15.2%) |  | 43(26.4%) | 43(26.4%) |  |
| Colon cancer | 66(38.4%) | 498(41.8%) |  | 63(38.7%) | 62(38.0%) |  |
| Rectal cancer | 58(33.7%) | 511(42.9%) |  | 57(35.0%) | 58(35.6%) |  |
| **aCCI** | 5.26±1.21 | 4.98±1.29 | 0.008 | 5.21±1.19 | 5.28±1.28 | 0.592 |
| **Smoking** | 63(36.6%) | 347(29.2%) | 0.046 | 60(36.8%) | 57(35.0%) | 0.729 |
| **Alcohol consumption** | 48(27.9%) | 273(22.9%) | 0.151 | 45(27.6%) | 48(29.4%) | 0.542 |
| **Hypertension** | 52(30.2%) | 318(26.7%) | 0.333 | 48(29.4%) | 50(30.1%) | 0.809 |
| **Diabetes** | 24(14.0%) | 168(14.1%) | 0.954 | 22(13.5%) | 24(14.7%) | 0.759 |
| **Chronic pulmonary disease** | 36(20.9%) | 159(13.4%) | 0.009 | 26(16.0%) | 31(19.0%) | 0.466 |
| **Coronary artery disease** | 13(7.6%) | 75(6.3%) | 0.531 | 12(7.4%) | 11(6.7%) | 0.829 |
| **Neoadjuvant radiotherapy** | 12(7.0%) | 80(6.7%) | 0.901 | 10(6.1%) | 12(7.4%) | 0.619 |
| **Neoadjuvant chemtherapy** | 28(16.3%) | 152(12.8%) | 0.204 | 25(15.3%) | 21(12.9%) | 0.525 |
| **Operative time (min)** | 224.17±77.84 | 206.00±73.84 | 0.003 | 222.14±73.36 | 222.25±79.62 | 0.715 |
| **Blood loss (ml)** | 93.14±101.02 | 77.99±96.77 | 0.057 | 91.72±98.23 | 96.60±107.77 | 0.670 |
| **Preoperative Hb (g/L)** | 117.86±23.65 | 120.68±23.50 | 0.148 | 117.30±23.76 | 118.40±26.66 | 0.696 |
| **Preoperative WBC (10^9^/L)** | 5.54±1.58 | 5.70±1.91 | 0.309 | 5.57±1.61 | 5.62±1.85 | 0.821 |
| **Preoperative Alb (g/L)** | 36.23±4.10 | 37.22±12.19 | 0.041 | 36.48±4.35 | 35.64±5.19 | 0.111 |
| **Preoperative IL-6 (pg/ml)** | 7.41±8.01 | 8.97±26.33 | 0.557 | 7.44±8.18 | 13.19±57.46 | 0337 |

PSM: Propensity Score Matching, ASA: American Society of Anesthesiologists, aCCI: Age-adjust Charlson Comorbidity Index, Hb: Hemoglobin, WBC: White Blood Cell, Alb: albumin, IL-6: interleukin-6
